# The effect of maternal exercise during pregnancy on abnormal fetal growth

**DOI:** 10.3325/cmj.2013.54.362

**Published:** 2013-08

**Authors:** Vlatka Tomić, Goran Sporiš, Jozo Tomić, Zoran Milanović, Djurdja Zigmundovac-Klaić, Saša Pantelić

**Affiliations:** 1 Department of Gynecology and Obstetrics, University Hospital Center “Sestre Milosrdnice,” Zagreb, Croatia; 2 Department of General and Applied Kinesiology, Faculty of Kinesiology, Zagreb, Croatia; 3 Faculty of Sport and Physical Education, University of Niš, Niš, Serbia; 4 Primary obstetrics and gynecology care center “DZ Zagreb Center,” Zagreb, Croatia

## Abstract

**Aim:**

To assess the effect of maternal physical activity during pregnancy on abnormal fetal growth.

**Methods:**

The study group of 166 women in gestational week 6-8 exercised regularly three days per week at submaximal intensity during their entire pregnancy and the control group of 168 women received standard antenatal care. The main outcomes were macrosomia and intrauterine growth restriction.

**Results:**

The study group had a lower frequency of macrosomia in newborns (6.0% vs 12.5%, *P* = 0.048) and gestational diabetes (1.8% vs 8.3%, *P* = 0.008) than the control-group, but there was no significant difference in intrauterine growth restriction (7.2% vs 6.5%). There was also no significant differences in other perinatal outcomes.

**Conclusions:**

The beneficial effect of maternal physical activity on fetal growth may be caused the impact of aerobic exercise on glucose tolerance. Fitness trainers and kinesiologists, as well as health care providers, should be educated on the benefits of regular exercise during pregnancy and safe physical exercise for pregnant women.

Intrauterine growth restriction (IUGR) and excessive birth weight are associated with numerous maternal and neonatal complications. Birth weight of over 4000 g causes higher incidence of postpartum hemorrhage, Cesarean sections, shoulder dystocia, birth traumas, and the risk of developing obesity and diabetes mellitus later in life (1,2). IUGR infants are at higher risk for complications of prematurity, including chronic lung disease and necrotizing enterocolitis (3). Childhood implications for IUGR include an increased risk of short stature, cognitive delays with decreased academic achievement, and neurological disorders, including cerebral palsy (4).

The incidence of macrosomia and IUGR together amounts to 15%-20% (5,6), causing serious public health consequences, especially in developing countries. Factors associated with fetal development include maternal socioeconomic status, marital status, blood pressure, diabetes mellitus, smoking, life style, and prenatal care (7,8). An important additional effect could also be maternal physical activity in pregnancy, positive (9,10), negative (11-13), and neutral (14,15) effects of which have been described. The vast majority of studies have investigated the impact of short-term exercise programs (of only a few days’ or a few weeks’ duration) and few studies investigated longer exercise programs, especially in the third trimester of pregnancy. The aim of this study was to assess the effect of regular exercise performed during all trimesters of pregnancy on abnormal fetal growth.

## Methods

### Study population and inclusion criteria

The study was conducted between July 2008 and December 2009 at the University Clinical Hospital “Sestre Milosrdnice,” University of Zagreb and corresponding primary care units. At their first obstetric and ultrasound examination (6-8 weeks of gestation), all women who fulfilled the inclusion criteria were offered participation in the study. Inclusion criteria were good general health, age 18-35 years, viable fetus at the regular ultrasound scan, and normal pregnancy confirmed by a clinical obstetrician or primary care obstetrician. Exclusion criteria were chronic or acute medical conditions (cancer; renal, endocrinologic, psychiatric, neurological, infectious, and cardiovascular diseases), multiple gestations, persistent second or third trimester bleeding, placenta previa after the 26th week of gestation, poorly controlled hypertension, diabetes mellitus or thyroid gland disease, as well as incompetent cervix, history of recurrent miscarriages, heavy smoking habit, and conditions that could interfere with ACOG (16) and ACSM (17) guidelines recommendations. The prenatal screening included assessment of women's general health provided by their family physician and obstetric history provided by their primary gynecologist. Three hundred and sixty pregnant women were divided into two groups depending on the ordinal number of their entry into the study, ie, every odd-numbered participant was included in the study group and every even-numbered participant into the control group. A total number of 166 women in the study group and 168 women in the control group completed the study from the start (6th-8th week of pregnancy) till the end (the week of delivery), which made it possible to meet the power-related demands of the study reflected in a moderate effect size. Eight women in the study group and 7 women in the control group left the program on their request; 6 more women in the study group and 5 women in the control group had pregnancy complications preventing them to further participate in physical exercise programs. The overall drop-out rate was 7.8% in the study group and 6.7% in the control group. The condition for involvement of participants in the study group was taking part in 80% of exercise sessions during the experimental program. Women gave their written informed consent before entering the study and institutional Ethics Committee approved the trial.

### Outcome measures

The primary outcome measure was the effect of regular aerobic exercise during pregnancy on abnormal fetal growth, namely, on intrauterine growth restriction (IUGR) and excessive fetal growth (macrosomia). IUGR is defined as a birth weight <10th percentile for the gestational age (18) and fetal macrosomia as birth weight >4000 g. Secondary outcomes were preeclampsia, pregnancy-induced hypertension, gestational diabetes, and mode of delivery. Gestational diabetes was determined at 24-28 weeks gestation using a 50 g glucose test. Blood glucose was measured one hour after glucose intake and the value of >140 mg/dL was considered significant, and diabetes was confirmed on glucose tolerance test (19). Pregnancy-induced hypertension was defined as persistently elevated blood pressure (diastolic blood pressure of 90 mm Hg or higher and systolic pressure of 140 mm Hg and higher on more than two occasions) with proteinuria or edema or both (19). Mode of delivery was defined as delivery before 37 completed weeks of gestation. Preeclampsia was defined as systolic blood pressure of 140 mm Hg or higher or diastolic blood pressure of 90 mm Hg or higher occurring after 20 weeks of gestation in a woman whose blood pressure had previously been normal according to ACOG Practice Guidelines (20).

### Intervention

The study group was engaged in regular aerobic exercise that consisted of a warm-up period (5 minutes), aerobic exercise (30 minutes), stretching (10 minutes), and the cool-down period (5 minutes). Exercise was performed 3 times per week during the whole pregnancy period. All sessions were supervised by an expert kinesiologist. The exercise program and intensity were in compliance with the guidelines set by the ACOG (16) and the ACSM (17).

The aerobic activities were performed at a moderate intensity assessed by the Rated Perceived Exertion Scale (6-19,21), and at 60%-75% of the maximum heart rate, controlled by a heart rate monitor (Polar Electro OY CE 0537, Kempele, Finland). Intensity, measured on the Borg Rated Perceived Exertion Scale, lower than 10 was considered to be low, between 11 and 14 moderate, and above 14 high (21). The control group comprised healthy pregnant women who did not participate in any organized regular physical exercise during pregnancy. The intensity of exercise was estimated by questionnaire to be between 2.5 Metabolic Equivalents of Task (METs) both for the introductory and the stretching period and up to 4 METs for the main part of the exercise session. The control group comprised healthy pregnant women who did not participate in any organized regular physical exercise during pregnancy. All control participants participated in everyday physical activities whose intensity was below 2.0 METs. Physical activity of controls included all activities performed in everyday life, in household and/or at workplace, which were also performed by participants from the experimental group. The controls were instructed not to engage in any kind of exercise except daily routine activities. These criteria ensured that the exercise program of the experimental group could be regarded as the main intervention measure.

### Follow-up

Women visited an obstetrician four times. The first visit in the early pregnancy (6th-8th week of gestation) included collecting information on women's general health, obstetric history, examination (physical and obstetric), and the confirmation of fetal vital signs by the ultrasound. The second visit, in the middle of the second trimester (20th gestational week), included regular obstetric examination and fetal anomaly scan ultrasound. The detailed physical examination included measurements of blood pressure, woman's weight gain, and urine screening test for albuminuria. Upon the confirmation of the normal course of pregnancy, the women were allowed to continue with their participation in the study. The third visit, in the middle of the third trimester (32nd gestational week), included physical examination, obstetric and ultrasound assessment, and noting of possible pregnancy complications. The fourth visit was 6 weeks postpartum, when the obstetric outcome was discussed and recorded from the clinics register file: pregnancy length, mode of delivery, and neonatal birth weight.

### Statistical analysis

Data analysis was conducted using the SPSS, version 17.0 (SPSS Inc., Chicago, IL, USA). Descriptive statistics were computed for all experimental data and separately for both groups and for all variables as mean ± standard deviation for quantitative, or count and percentage for categorical variables. In addition, Kolmogorov-Smirnov test was used to test the normality of distribution before further analysis. Differences between the groups were determined using the χ^2^ test and the t*-*test (age, body mass index, gestational age in weeks, birth weight in grams, birth length in centimeters). Correlations between the intensity of training and the outcome variables were determined using Spearman correlation coefficient. Statistical significance was set at the level of *P* < 0.05.

## Results

Kolmogorov-Smirnov test showed that all variables had normal distribution (*P* < 0.05). The groups did not significantly differ in age, pre-pregnancy body mass index, and smoking status (Table 1). There was 45.2% nulliparous women in the study group and 35.7% in the control group (Table 1). More than 70% of all participants had secondary education (the duration of education totaling 12 years), 20% had a college or university degree, and 5% had primary education (<9 years), with non-significant differences between the groups. The majority of our participants were married; 86.1% in the study group and 83.9% in the control group.

**Table 1:** Characteristics of mothers who performed physical exercise during pregnancy and those who did not

	Study group (N = 166)	Control group (N = 168)	*P*
**Age**	28.9	29.2 ± 3.2	0.568
**Body height in cm (mean ± standard deviation)**	167.5 ± 6.0	168.2 ± 6.3	0.235
**Body mass in kg (mean ± standard deviation):**			
before pregnancy	64.8 ± 13.4	64.4 ± 11.1	0.769
at the end of pregnancy	78.2 ± 13.1	80.1 ± 11.9	0.180
**Body mass index in kg/m^2^ (mean ± standard deviation):**			
before pregnancy	23.1 ± 4.1	22.8 ± 4.5	0.547
at the end of pregnancy	27.9 ± 4.1	28.3 ± 3.7	0.352
**Borg scale (mean ± standard deviation) (**21**)**	11.9 ± 1.1	–	–
**Smoking (n, %)**	37 (22.3)	39 (23.2)	0.840
**Parity (n, %):**			
0	75 (45.2)	60 (35.7)	0.345
1	67 (40.4)	77 (45.8)	0.467
2	18 (10.8)	25 (14.9)	0.675
≥3	6 (3.6)	6 (3.6)	0.589
**Education (n, %):**			
<9 y	5 (3.0)	7 (4.2)	0.385
11-12 y	121 (72.9)	128 (76.2)	0.457
12-16 y	11 (6.6)	13 (7.7)	0.386
>16 y	29 (17.5)	20 (11.9)	0.649
**Marital status (n, %):**			
married	143 (86.1)	141 (83.9)	0.439
not married	23 (13.9)	27 (16.1)	0.371

Gestational diabetes and excessive newborns’ weight occurred significantly more frequently in the control than in the study group (Table 2). The mean birth weight was also higher in the control group, but not significantly (3374.3 g vs 3292.1 g). There was no significant difference between the groups in birth length, IUGR, preterm birth, pregnancy-induced hypertension, preeclampsia, and mode of delivery.

**Table 2:** Regular aerobic exercises and pregnancy outcome

	Nulliparous		*P*	Multiparous		*P*	Total		*P*
	study group n = 75	control group n = 60		study group n = 91	control group n = 108		study group n = 166	control group n = 168	
**Pregnancy induced hypertension (n, %)**	1 (1.3)	0 (0.0)	0.321	3 (3.3)	5 (4.6)	0.483	4 (2.4)	5 (2.8)	0.739
**Gestational diabetes (n, %)**	2 (2.7)	4 (6.7)	0.414	1 (1.1)	10 (9.3)	0.011*	3 (1.8)	14 (8.3)	0.008*
**Preeclampsia (n, %)**	0 (0.0)	1 (1.7)	0.319	1 (1.1)	1 (0.9)	1.000	1 (0.6)	2 (1.2)	0.564
**Gestational age in weeks** (mean ± standard deviation)	38.7 ± 2.4	39.4 ± 1.3	0.061	38.9 ± 1.7	38.9 ± 2.1	0.818	38.8 ± 0.4	39.1 ± 0.6	0.346
**Preterm birth (n, %)**	8 (10.7)	6 (10.0)	0.594	6 (6.6)	3 (2.8)	0.307	14 (8.4)	9 (5.4)	0.297
**Birth weight in grams**	3289.6 ± 610.5	3410.2 ± 425.5	0.203	3294.2 ± 470.3	3354.4 ± 609.3	0.441	3292.1 ± 536.5	3374.3 ± 550.1	0.214
**Birth length in centimeters**	49.2 ± 3.1	49.9 ± 2.0	0.151	49.4 ± 2.4	49.6 ± 3.0	0.563	49.3 ± 0.2	49.7 ± 0.3	0.321
**Intrauterine growth retardation (n, %)**	7 (9.3)	4 (6.7)	0.372	5 (5.5)	7 (6.5)	0.557	12 (7.2)	11 (6.5)	0.835
**Excessive newborns weight (n, %)**	7 (9.3)	10 (16.7)	0.469	3 (3.3)	11 (10.2)	0.031*	10 (6.0)	21 (12.5)	0.048*
**Vaginal delivery (n, %)**	62 (82.7)	46 (76.7)	0.124	77 (84.6)	90 (83.3)	0.306	139 (83.7)	136 (81.0)	0.856
**Cesarean section (n, %)**	13 (17.3)	14 (23.3)	0.843	14 (15.4)	18 (16.7)	0.484	27 (16.3)	32 (19.0)	0.515

*Statistically significant differences, *P* < 0.05.

The relationship between pregnancy outcome and perceived intensity of the exercise was not significant (*P* > 0.05; Table 3). The maximum reported value on the scale was 14 (equivalent to 75% of the effort exerted to accomplish a certain physical activity); therefore the vigorous exercise program was not applied due to possible damage it could cause to mothers and children, according to ACOG recommendations.

**Table 3:** Relationship between pregnancy outcome and intensity of regular aerobic exercise

	Borg scale (21)		
	<10 (very light)	11-13 (moderate)	>13 (intensive)
**Pregnancy induced hypertension**	0.14	0.19	0.21
**Gestational diabetes**	0.17	0.14	0.18
**Preeclampsia**	0.21	0.15	0.15
**Gestational age (weeks)**	0.18	0.17	0.22
**Preterm birth**	0.19	0.14	0.23
**Birth weight (g)**	0.18	0.16	0.17
**Birth length (cm)**	0.17	0.28	0.21
**Intrauterine growth retardation**	0.16	0.14	0.22
**Excessive newborns weight (>4000g)**	0.12	0.16	0.18
**Vaginal delivery**	0.14	0.18	0.16
**Cesarean section**	0.15	0.12	0.15

*Data are presented as *r*-values.

## Discussion

In this study, pregnant women who engaged in regular aerobic exercise during their pregnancy showed lower occurrence of fetal macrosomia (>4000 g) and gestational diabetes than those who did not.

The strengths of this study are the prospective design and controlled frequency, duration, and intensity of physical activity. The limitations are a relatively small sample size for some variables, which reduces the capacity to detect the differences in some more rare events concerning pregnancy outcome.

The relationship between physical activity during pregnancy and mean birth weight was extensively studied, but the results are contradictory (9-15). Recent reviews found benefits of exercise in pregnancy, despite methodological problems in some studies (22,23). These reviews concluded that further research is needed to fill in these gaps and to provide guidelines on intensity, duration, and frequency of physical activity participation during pregnancy.

The difference in mean birth weight may be of little clinical relevance to obstetricians since the extreme birth weight-related values (IUGR and macrosomia) are a risk factor for maternal and fetal complications. However, little is known about the effects of physical activity on fetal development. Spinillo et al (24) concluded that pregnant women who participated in moderate-to-high intensity occupational physical activities had a significantly higher risk of IUGR than women who participated in low intensity activities. Other authors did not find an increased risk related to low and moderate physical activity (10,14,15).

Our results confirm such findings. Participation in moderate-intensity aerobic exercise did not show any increased risk of IUGR and we did not investigate participation in high-intensity physical activities. Several studies have found a reduced frequency of preeclampsia (PE) and pregnancy-induced hypertension (PIH) in women who participated in low- and moderate-intensity physical activities (25-27). Since PIH and PE are both well-known risk factors for the development of IUGR, it is reasonable to believe that participation in low-to-moderate intensity physical activities would decrease the incidence of IUGR.

The protective effect of regular aerobic exercise participation on macrosomia observed in our study is in agreement with the epidemiological study by Owe et al (28). However, one recent study (29) did not show any either beneficial or harmful effect of physical activity participation during pregnancy on macrosomia. The differences between these two studies could be a consequence of different study designs and types, as well as intensity and frequency of regular physical exercise participation during pregnancy. Also, both studies used questionnaires for measuring physical activity participation, which have limited accuracy and precision in assessing exercise duration and intensity (30).

The beneficial effect of maternal physical activity participation during pregnancy on fetal growth may be an impact of aerobic exercise on glucose tolerance. Dempsey et al (31) found that physically active pregnant women had a lower risk of gestational diabetes than sedentary pregnant women. Physical activity participation during pregnancy may reduce the glucose level in women with gestational diabetes and in non-diabetic pregnant women (32). The findings of our study, ie, the reduced occurrence of gestational diabetes in pregnant women who perform regular aerobic exercise, support the result of such studies.

In conclusion, submaximal aerobic exercise program participation was not found to be associated with abnormal fetal growth, and the findings of this study even imply possible beneficial effects of regular aerobic exercise participation during pregnancy. However, there is a need for further studies with reports on different frequency, duration, and intensities of physical activity before making any definitive conclusion regarding beneficial effects on fetal growth and pregnancy outcome.

Maternal physical activity participation in moderate-intensity aerobic exercise did not carry any increased risk of IUGR. The beneficial effect of maternal physical activity on fetal growth may be caused by the impact of aerobic exercise on glucose tolerance. Fitness trainers and kinesiologists, as well as health care providers, should be educated on the benefits of regular exercise during pregnancy and safe physical exercise for pregnant women.
